# Stigma and Endometriosis: A Brief Overview and Recommendations to Improve Psychosocial Well-Being and Diagnostic Delay

**DOI:** 10.3390/ijerph18158210

**Published:** 2021-08-03

**Authors:** Omar T. Sims, Jhumka Gupta, Stacey A. Missmer, Irene O. Aninye

**Affiliations:** 1 Department of Social Work, College of Arts and Sciences, University of Alabama at Birmingham, Birmingham, AL 35294, USA; sims.omar@gmail.com; 2 Department of Health Behavior, School of Public Health, University of Alabama at Birmingham, Birmingham, AL 35294, USA; 3 Center for AIDS Research, School of Medicine, University of Alabama at Birmingham, Birmingham, AL 35294, USA; 4 Integrative Center for Healthy Aging, School of Medicine, University of Alabama at Birmingham, Birmingham, AL 35294, USA; 5 African American Studies, College of Arts and Sciences, University of Alabama at Birmingham, Birmingham, AL 35294, USA; 6 Department of Global and Community Health, College of Health and Human Services, George Mason University, Fairfax, VA 22030, USA; jgupta4@gmu.edu; 7 Department of Obstetrics, Gynecology and Reproductive Biology, College of Medicine, Michigan State University, East Lansing, MI 49503, USA; stacey.missmer@hc.msu.edu; 8 Department of Epidemiology, Harvard T.H. Chan School of Public Health, Harvard University, Cambridge, MA 02115, USA; 9 Society for Women’s Health Research, Washington, DC 20036, USA

**Keywords:** endometriosis, stigma, diagnostic delay, gynecological disease, chronic pelvic pain, women’s health, psychosocial well-being

## Abstract

Endometriosis is a chronic gynecological disease that affects approximately 1 in 10 women of reproductive age. Symptoms of severe pelvic pain, infertility, fatigue, and abnormal menstruation can cause significant negative effects on an individual’s physical and mental health, including interactions with their family, friends, and health care providers. Stigma associated with endometriosis has been under-studied and is rarely discussed in current literature. Herein, this paper aims to provide a brief overview of published literature to explore and establish the plausibility of stigma as a driver of suboptimal psychosocial well-being and diagnostic delay among individuals living with endometriosis. We present the clinical characteristics and physical and mental health consequences associated with endometriosis, highlight several theoretical constructs of stigma, and review the limited studies documenting women’s lived experiences of endometriosis-related stigma. To mitigate harmful effects of this phenomenon, we recommend increasing efforts to assess the prevalence of and to characterize endometriosis-related stigma, implementing awareness campaigns, and developing interventions that combat the multidimensional negative effects of stigma on timely care, treatment, and quality of life for individuals living with endometriosis.

## 1. Introduction

Endometriosis is an inflammatory and often painful chronic gynecological condition characterized by the presence of tissue similar to the uterine lining (endometrium) outside of the uterus, mainly occurring in the pelvic peritoneum, rectovaginal septum, ovary, uterosacral ligament, rectum, or colon [1,2,3,4]. Ectopic tissue can form lesions in these areas, leading to chronic pain and other debilitating symptoms [1]. Endometriosis has wide-ranging and pervasive sequelae on women’s health (e.g., physical and mental health) [5].

The Society for Women’s Health Research convened an interdisciplinary expert group of researchers, clinicians, patients, and policy leaders for a roundtable discussion to review current clinical practice, reflect on the barriers affecting diagnosis and treatment, and highlight research priorities for the future of endometriosis care [6]. Several topics emerged from the roundtable, but notably, the working group arrived at consensus that endometriosis-related stigma and its impact on psychosocial well-being are barriers that contribute to diagnostic delay (i.e., the delayed time between the onset of symptoms and diagnosis or treatment). Surprisingly, endometriosis-related stigma has been under-studied and is rarely discussed in current literature.

Herein, we aimed to provide a brief overview of published literature to explore and establish the plausibility of stigma as a driver of suboptimal psychosocial well-being and diagnostic delay among individuals living with endometriosis. The objectives of this paper are to briefly highlight constructs of stigma, to characterize endometriosis-related stigma and the ways in which it impacts psychosocial well-being and diagnostic delay, and to propose next steps to improve research and health care on this topic. To achieve these goals, we briefly describe endometriosis epidemiology, clinical characteristics, and physical and mental health consequences of the disease. We highlight several theoretical constructs of stigma, illuminate women’s lived experiences of endometriosis-related stigma found in published literature, and proffer recommendations for next steps in research, public health, and clinical care to move the field forward in understanding and mitigating harmful effects of endometriosis-related stigma.

## 2. Epidemiology, Natural History and Comorbidities

The prevalence of endometriosis among reproductive-aged women is estimated to be about 10%, representing 190 million women worldwide [4]. Globally, years lived with disability (YLDs) and disability-adjusted life years (DALYs) due to endometriosis were 56.5 (with a 95% uncertainty interval of 34.0–89.6) and 56.6 (35.0–89.7) per 100,000 in 2019, respectively [7]. In a recent meta-analysis of 69 studies conducted in a variety of countries [8], the pooled prevalence of endometriosis by gynecologic indication was highest among women with chronic pelvic pain (47%), followed by infertility (34%), hysterectomy (22%), ovarian cancer (12%), and tubal sterilization (11%). Furthermore, prevalence varied across continents, with Asia having the highest prevalence of endometriosis (21%), followed by the Americas (North and South, 13%), Europe (12%), Africa (11%), and Australia (4%).

Although the natural history and pathophysiology of endometriosis are not fully understood [9], it is well-established that symptoms of endometriosis can be progressive and recurrent. These symptoms include, but are not limited to, cyclic pelvic pain (dysmenorrhea), acyclic pelvic pain that can be chronic (lasting more than six months), painful vaginal intercourse (dyspareunia), painful urination (dysuria), painful bowel movements (dyschezia), infertility, fatigue, heavy menstrual bleeding, and intermenstrual bleeding [1,2,4,10,11,12], with greater risk of pain-associated conditions such as fibromyalgia, rheumatoid arthritis [13], and migraine [14]. Among those successfully diagnosed, chronic pelvic pain and infertility are the most common presenting symptoms associated with endometriosis [10,15,16,17].

A sizable number of large population-based, case–control, tertiary studies (i.e., systematic reviews and meta-analyses) have demonstrated that gastrointestinal (e.g., irritable bowel syndrome and inflammatory bowel disease), immunological (e.g., rheumatoid arthritis and psoriasis), and cardiovascular (e.g., coronary heart disease, hypercholesterolemia, and hypertension) diseases co-occur at greater rates in women with endometriosis than those without endometriosis [18,19,20]. Although endometriosis is a non-malignant condition, a substantive body of evidence suggests endometriosis is associated with increased risk for cancer, particularly ovarian and breast cancers [20,21].

There are four primary types of endometriosis: subtle (polypoid- and flame-like lesions and lesions with red and white vesicles), typical (burnt-out powder pigmented lesions), cystic (brown, tar-like ovarian lesions), and deep (lesions deeper than 5mm under the peritoneum) [22]. Subtle and typical are the most common types of endometriosis—most prevalent among women with chronic pelvic pain or infertility (80% and 50%, respectively)—followed by cystic (25%) and deep (5%) endometriosis. Through laparoscopy and histological confirmation, surgical staging of endometriosis takes into account the location, extent, and depth of lesions on a cumulative scoring system: Stage I (minimal, 1–5 points), Stage II (mild, 6–15 points), Stage III (moderate, 16–40 points), and Stage IV (severe, >40 points) [1,23,24]. Staging does not necessarily take into account endometriosis outside of the pelvic region, and it is utilized more to assess reproductive potential than the severity of symptoms or the impact on quality of life. For example, an individual with Stage IV endometriosis may experience fewer life-disrupting symptoms than someone with Stage I disease.

Risk factors for endometriosis are heterogeneous. Early age at menarche (before 11–13 years of age), short menstrual cycle length (≤27 days), longer menstrual flow (≥7 days), and family history of endometriosis among first-degree relatives (e.g., mother or sister) are associated with increased risk for endometriosis [25,26]. Lower body mass index and high consumption of red meats and trans fats have also been noted as risk factors [26,27]. On the other hand, oral contraceptive use, physical activity, and a diet high in fruits, greens, vegetables, and omega-3 long-chain fatty acids have been shown to decrease risk for endometriosis [28,29]. Notably, studies that have characterized risk factors for and consequences of endometriosis lacked sufficient inclusion of women of color. More robust and inclusive studies are needed to determine whether risk factors differ for diverse populations of women [30,31,32] or for specific subtypes of endometriosis [4,33].

## 3. Physical and Mental Health Consequences of Endometriosis

There are significant physical and mental health consequences of endometriosis. Chronic menstrual and non-menstrual acyclic pain and other symptoms associated with endometriosis have been reported to negatively impact and limit daily life activities and work performance [34,35,36]. Many people with endometriosis report an inability to complete or engage in day-to-day tasks, household chores, childcare-related activities, and physical exercise. Across multiple studies, participants also reported decreases in the quality of their work, significant limitations in their ability to perform work-related activities without disruption, and 11 to 19 days of missed work per year [34,37,38,39].

The negative effects of endometriosis on psychosocial well-being, which can start as early as adolescence [38,40,41], have been assessed and well-documented by a mix of quantitative, qualitative, and tertiary studies. These effects include increased emotional and psychological distress, decreased emotional well-being, reduced quality of sleep, diminished health-related and overall quality of life, and higher prevalence and severity of symptoms of depression and anxiety [37,42,43,44,45,46,47,48]. Individuals with endometriosis can experience adverse effects on sexual function and satisfaction, such as painful intercourse, avoidance of intercourse, lower levels of desire and arousal, and poorer orgasm [49,50,51]. Additional impacts include strained intimate and social relationships (e.g., relationship conflict and divorce) and more frequent avoidance or withdrawal from social engagement [51,52]. Altogether, psychosocial well-being of individuals living with endometriosis is considerably lower than those without endometriosis.

Though overlooked in published literature, stigma may be an underlying social phenomenon that catalyzes diminished psychosocial well-being among individuals living with endometriosis. Stigma—whether internalized or in familial, intimate, work, and health care settings—has the potential to exacerbate the aforementioned negative effects of endometriosis and sustain poor psychosocial well-being. Equally important, endometriosis-related stigma can be a barrier to timely diagnosis. Mean estimates of diagnostic delay range between 7 and 11 years from onset of symptoms to diagnosis of endometriosis, and the consequences may include increased symptom and disease severity, exacerbation of physical and psychosocial sequelae, and late access to effective treatment and care [38,53,54].

## 4. Stigma

Stigma is a complex construct that has been conceptualized and described by scholars from various disciplines. Goffman’s early work on stigma is widely considered seminal in stigma research [55]. His definition of stigma is composed of an attribute or characteristic that is deemed socially undesirable and of two groups of people: those who stigmatize and those who are stigmatized. Goffman refers to stigma as a stain, mark, or attribute that people may deem as socially undesirable and, in turn, use the attribute to set themselves apart from others. Stigmatization is defined as the act or behavior that disqualifies a person from full social acceptance, and it reduces them to a tainted, discounted person.

Other definitions of stigma and stigmatization have materialized that expand on Goffman’s definition. Link et al. 2001 conceptualizes stigma as the exercise of power to simultaneously label, stereotype, and cause separation, discrimination, and loss of status in the lives of others [56]. Additionally, definitions include a pattern or habit of judging and excluding others as illegitimate interactants [57], and an attribute or characteristic that is devalued by others in particular social settings [58].

Scambler 1998 views stigma as a layered phenomenon: enacted stigma and felt stigma [59]. Enacted stigma refers to the experience of unfair, unjustifiable, and discriminatory treatment by others; whereas, felt stigma refers to the experience of anticipatory or actual shame that prevents people from disclosing their negative experiences and inhibits their willingness or ability to seek help. Oftentimes, felt stigma leads to self-stigmatization (i.e., incorporation or acceptance of others’ prejudices and stereotypes into beliefs about oneself) [60]. Phelan et al. 2008 describes three characteristics of stigma: keeping people down (exploitation and domination), keeping people in (enforcement of social norms), and keeping people away (avoidance of disease) [61].

Similarly, stigma not only has the potential to lead to societal and self-devaluation, but it can also adversely impact health. Stuber et al. 2008 proposes that social interactions between marginalized and non-marginalized groups are health-harming to the former [62]. Segregation efforts initiated by non-marginalized groups toward marginalized groups lead to poor health outcomes due to denial or limited access to similar socioeconomic and health resources. Furthermore, anticipation of stigma (perceived or actual stigma) or unfair treatment can lead to various forms of psychological distress that inhibits optimal wellness and well-being.

Hatzenbuehler et al. 2013 argues that stigma is a central driver of social determinants of health and of morbidity and mortality [63]. Stigma mediates, undermines, and corrodes psychosocial well-being (e.g., social relationships, social support, self-worth, and quality of life). Over time, continued exposure to unfair treatment inherent in stigma compromises psychological fortitude, which leads to adverse or suboptimal physical and mental health outcomes, such as psychological distress, anxiety, and/or depression.

Stigma has been widely assessed and examined in studies on various health and psychiatric conditions: human immunodeficiency virus (HIV), hepatitis C virus (HCV), obesity, major depressive disorder, generalized anxiety disorder, bipolar disorder, alcohol and substance use, and type 2 diabetes mellitus (T2DM) [64,65,66,67,68,69,70,71,72,73]. Respective studies have demonstrated that stigma is commonly experienced by people with specific health and psychiatric conditions, and that stigma is negatively associated with multiple outcomes: suboptimal health or help-seeking behaviors, low engagement in health care, low treatment-seeking behaviors, and low testing or diagnostic uptake. Together, these stigma-driven outcomes lead to barriers to care and treatment—both underdiagnosis and diagnostic delay. Similarly, endometriosis-related stigma may deteriorate psychosocial well-being and lead to diagnostic delay.

## 5. Endometriosis-Related Stigma

Matias-Gonzalez et al. 2020 conducted a qualitative study to explore the experiences of stigma among Latina women living with endometriosis [74]. Convenience sampling was used to recruit participants from an endometriosis patient advocacy foundation in Puerto Rico. During focus group discussions, researchers used a semi-structured interview guide to illicit participants’ experiences, opinions, perceptions, and beliefs on living with endometriosis and endometriosis-related stigma.

The primary emergent theme from the analysis of the interviews was endometriosis as *changueria*, a term used to label or describe someone as being an excessive whiner or complainer without an apparent reason. Throughout the focus groups, participants stated that they were labeled by others as *changas* in three distinct interactions: family interactions, health care interactions, and social interactions.

Participants reported that their spouses and family members minimized or dismissed their unbearable pain associated with endometriosis because they perceived pain during menstruation to be something normal for women to endure. Participants felt as if they were expected to endure endometriosis-related pain and to not allow it to interfere or limit their everyday lives, social activities, and familial responsibilities. Participants stated that their spouses and family members perceived them as “putting on a show” and exaggerating pain symptoms. They also expressed feelings of rejection because spouses and family members were unwilling to recognize that their pain severity and experiences were both legitimate and debilitating.

In health care settings, participants articulated experiences of being labeled as *changas* by health care providers when they mentioned their pain symptomatology during visits. Participants reported experiences of providers laughing at their pain symptoms and, at times, explaining in a condescending manner what participants were supposedly experiencing (e.g., gas or bloating) without further diagnostic investigation. This suggests both normalization of symptoms (saying there is nothing wrong) and invalidation (rejecting or denying someone’s emotions and experiences).

Participants stated that endometriosis is perceived by the general public as less serious than other chronic medical conditions. They also said that the portrayal of women in the media reinforces a false notion that women should “not let their period prevent [them] from doing anything.” The authors concluded that endometriosis-related stigma is an important but underappreciated psychosocial phenomenon. Exposure to continued experiences of dismissiveness, non-conforming repercussions, invalidation, and poor understanding by others in family, health care, and social interactions contributes to negative coping and symptomatology concealment, which then leads to diagnostic delay.

Seear et al. 2009 conducted a qualitative study in Australia to explore the experiences of women living with endometriosis [75]. Researchers recruited participants using snowball and convenience sampling, conducted individual, semi-structured interviews, and identified emergent themes. Themes of symptom concealment and adhering to “menstrual etiquette” to avoid stigmatization from people in familial, social, and health care settings emerged from the analysis. In instances when participants disclosed chronic pelvic pain, heavy bleeding, or painful menstruation associated with their endometriosis, their subjective experiences of the debilitating symptoms were redefined or normalized by others as a “normal load in life” that women were expected to carry and deal with until menopause. Trivialization of symptoms and being viewed by others as having poor menstrual etiquette led to participants masking their painful symptomatology.

Participants reported repercussions related to violations of menstrual etiquette or symptom disclosure that reinforced stigma associated with endometriosis. They described being ostracized and criticized by family members, sexual partners, and employers. They stated that employers accused them of malingering, questioned the legitimacy of their symptoms, and issued reprimands. Their symptoms were not taken seriously because menstrual pain often is not a visible problem to others. When participants breached menstrual etiquette by disclosing their pain complaints before or during sex, their sexual partners would falsely perceive them as making excuses to withdraw from sex, resulting in participants often concealing their pain to avoid stigma associated with being a “cop out”. Feelings of “just my cross to bear and maybe I have to just suffer in silence” also translated to participants’ reluctance to report symptoms of chronic pelvic pain to health care providers.

Participants in Seear’s study also discussed experiences and feelings of ridicule and mockery by other women, as well as encouragement from other women (e.g., mothers) to not discuss or disclose complaints around menstrual pain and irregularities. One participant stated that mothers were likely raised to believe that painful menstruation was “normal” or “natural” and that “part of that was that [her mother] had always had very difficult periods when she was young but that there was no option, [so] it was never taken that seriously”.

The authors concluded disclosure of menstrual pain and irregularities or violations of menstrual etiquette culminated in sanctions, criticism, and ostracism that lead to behavioral changes in symptomatology disclosure to circumvent stigmatization. The authors also concluded that stigmatization hampered the willingness to act on abnormal symptoms because participants were socialized to think that abnormalities were normal for women. Overall, the authors asserted that endometriosis-related stigma is an emerging factor associated with diagnostic delay and that lack of awareness about endometriosis among women, their families, and intimate partners, as well as health care providers and the general population, perpetuates stigmatization and its negative effects on health and psychosocial well-being.

## 6. Recommendations

Based on our overview of the limited published literature on endometriosis and individuals’ lived experiences with this disease, we provide recommendations in research, clinical care, and education to address gaps in understanding and mitigating harmful effects of endometriosis-related stigma.

(1) *Assessment of the Prevalence of Endometriosis-Related Stigma*. Despite the high prevalence of endometriosis, there is only a small handful of qualitative studies exploring the phenomenology of endometriosis-related stigma. To date and to the authors’ knowledge, there are no published studies that have quantitatively assessed and established this phenomenon; therefore, while the frequency and extent of endometriosis-related stigma is unknown, we hypothesize that is likely a common occurrence. Repeated cross-sectional, longitudinal, and a variety of other study designs could be employed to estimate point prevalence, period prevalence, lifetime prevalence, and other epidemiologic parameters of endometriosis-related stigma. Such studies may also provide much needed data on life-course implications of stigma from adolescence to postmenopause.
(2) *Conceptualization and Development of Scales to Assess and Characterize Endometriosis-Related Stigma*. There are currently no published scales designed to assess stigma associated with endometriosis. Conceptualization and development of scales with validated psychometric properties to measure and assess constructs of endometriosis-related stigma are needed. These scales could be used to facilitate assessment of stigma in the general population and in clinical settings, such as primary care, adolescent medicine, obstetrics and gynecology, and women’s health clinics [41]. A number of scales have been developed, tested, and validated to measure and assess stigma experienced by those living with several medical conditions and psychiatric disorders such as HIV, HCV, obesity, tuberculosis, T2DM, depression, and anxiety [76,77,78,79,80]. These respective scales have advanced public health and clinical management of condition-specific stigma and improved awareness of stigma-related impacts in research and in clinical settings. The creation of endometriosis-specific scales could facilitate achievement of similar endpoints concerning endometriosis.

Scale development efforts should consider the amalgamation of theory-driven underpinnings of stigma and those that consistently emanate from qualitative studies exploring individuals’ lived experiences of endometriosis-related stigma. After psychometric testing and refinement, reliable and validated measures of endometriosis-related stigma should be publicly accessible to researchers and health care providers. Public health researchers could incorporate these scales in epidemiologic study designs to estimate and characterize the prevalence of endometriosis-related stigma, to identify potential sociodemographic disparities between patients with and without heavy burden of endometriosis-related stigma, and to examine and ascertain an array of epidemiologic associations. Health care providers could employ endometriosis-related stigma scales to identify patients in need of intervention, quantify patients’ severity of stigma burden, and assess intervention outcomes. In short, development of an endometriosis-related stigma scale has the potential to push the field forward in epidemiologic characterization, clinical assessment, and treatment outcomes research.

(3) *More Qualitative Studies on Endometriosis-Related Stigma*. There is a growing body of literature that examines stigma associated with menstruation and a smaller set that examines the lived experience of endometriosis concerning medical support and patient satisfaction; however, the number of studies with a specific focus on endometriosis-related stigma is meager. An in-depth search of databases (e.g., PubMed, PsychInfo, and CINAHL Plus) for published studies with “endometriosis” and “stigma” or “stigmatisation” in the title or abstract (publication range, 1980–April 2021) only yields five relevant publications: two qualitative studies on women’s lived experiences with endometriosis (presented in-depth in this paper) [74,75], one brief editorial [47], SWHR’s 2019 review paper on endometriosis [6], and a qualitative study on the perceptions of adolescent girls and boys about the symptoms of endometriosis as sources of stigma and shaming [41]. This is disconcerting, given the prevalence of endometriosis and its public health impact. More qualitative studies are needed to examine endometriosis-related stigma and to give voice to the experiences of individuals living with endometriosis—especially diverse groups of people.

Phenomenological, narrative, and other qualitative approaches can be used to create a safe space for individuals to disclose, reflect, and discuss their experiences with endometriosis and related stigma. Patterns and themes that materialize from individual and focus group semi-structured interviews can be used to further understand pathways among endometriosis, endometriosis-related stigma, psychosocial well-being, and diagnostic delay, as well as unknown pathways through which endometriosis-related stigma may influence physical, psychosocial, and other health outcomes. These findings could be used by public health professionals, health systems, and health care providers to inform intervention development and give credence to the provision of much-needed resources.

(4) *Widespread Endometriosis Awareness Campaigns*. Undoubtedly, targeted public health awareness campaigns improved awareness of HIV, HCV, T2DM, and mental illness among health systems, health care providers, and the general population [81,82,83,84,85,86,87,88]. They also modified negative societal beliefs and attitudes toward their respective marginalized populations. Implementing comparable awareness campaigns could achieve similar outcomes. Population-wide and health system-wide endometriosis awareness campaigns would foster a safe environment for education and healthy communication between individuals living with endometriosis and their family members, intimate partners, and health care providers [41]. Societal awareness and acknowledgment of endometriosis could increase empathy and support for those living with endometriosis, promote disclosure of symptoms among affected individuals, and result in more investigative responses by health care providers, which altogether may lead to earlier detection and treatment of endometriosis.
(5) *Development and Implementation of Anti-Stigma Interventions for Endometriosis-Related Stigma*. Findings from quantitative and qualitative studies could be used to develop and implement novel, theory-driven anti-stigma or stigma-reduction interventions. Alternatively, using implementation science approaches, existing interventions used to combat stigma for diseases such as HIV, HCV, and T2DM could be adapted for translation, testing, and use for endometriosis-related stigma. Endometriosis-related stigma is dynamic and multidimensional (e.g., intrapersonal, interpersonal, structural), thus, it is important to consider development and implementation of interventions with multilevel approaches [89]. Further, interventions should not be limited to delivery among the stigmatized. Whether a novel intervention or the translation of existing ones, approaches concerning endometriosis-related stigma should give equal consideration to the importance of engaging the stigmatized and stigmatizers, as well as the health systems, providers, and social environments that perpetuate stigmatization.

Intrapersonal intervention approaches are needed to negate the consequences of self-internalized endometriosis-related stigma. These approaches can also combat harmful and discriminatory attitudes held by those who are not stigmatized but who engage in stigmatization. Interpersonal interventions are needed to improve coping and health- and help-seeking behaviors among those who are stigmatized, while helping stigmatizers become aware of the adverse impact of both intentional and unintentional stigmatizing behaviors toward individuals living with endometriosis. Finally, structural intervention approaches are needed to improve awareness of endometriosis in the general population and across health systems. Awareness campaigns that highlight incidence, natural history, normal vs. abnormal symptoms, clinical characteristics, and debilitating consequences of endometriosis have the potential to reduce the frequency of stigmatization by directly and indirectly acknowledging the existence of this health reality.

(6) *Acknowledgement of and Stances against Endometriosis-Related Stigma by Professional Associations*. Professional associations in public health, medical, and women’s health spaces should strategically underscore and highlight endometriosis as a commonly deleterious yet overlooked health condition. Organizations have captive audiences for which public acknowledgement of endometriosis-related stigma as a factor that undermines physical and psychosocial well-being and access to adequate health care would have wide-reaching benefits. These organizations should also publicly take a stance against endometriosis-related stigma and issue a call for action among their relevant constituents. Such public actions could help widen the impact of novel awareness campaigns and encourage increased funding for public health and clinical intervention research for endometriosis-related stigma.

## 7. Conclusions

Despite the high prevalence and well-documented impacts of endometriosis on public health, stigma associated with this condition is vastly understudied. This article highlighted constructs of stigma and characterized endometriosis-related stigma and the ways in which it may contribute to psychosocial well-being and diagnostic delay. Without increased efforts to fully elucidate, understand, and estimate the quantitative extent and qualitative complexity of endometriosis-related stigma, individuals living with endometriosis-related stigma will continue to be affected by its potentially far-reaching and multidimensional negative effects on timely care, treatment, and quality of life. Hopefully, the recommendations articulated in this paper to quantify and address endometriosis-related stigma, research, and care will catalyze additional dialogue and provide insight for those who treat and care for individuals living with endometriosis.

## Data Availability

Not applicable.

## References

[1] Zondervan K.T., Becker C.M., Koga K., Missmer S.A., Taylor R.N., Viganò P. (2018). Endometriosis. Nat. Rev. Dis. Prim..

[2] Kiesel L., Sourouni M. (2019). Diagnosis of endometriosis in the 21st century. Climacteric.

[3] U.S. Department of Health and Human Services: Endometriosis. https://www.nichd.nih.gov/health/topics/endometriosis.

[4] Zondervan K.T., Becker C.M., Missmer S.A. (2020). Endometriosis. N. Engl. J. Med..

[5] Agarwal S.K., Chapron C., Giudice L.C., Laufer M.R., Leyland N., Missmer S.A., Singh S.S., Taylor H.S. (2019). Clinical diagnosis of endometriosis: A call to action. Am. J. Obstet. Gynecol..

[6] As-Sanie S., Black R., Giudice L.C., Gray Valbrun T., Gupta J., Jones B., Laufer M.R., Milspaw A.T., Missmer S.A., Norman A. (2019). Assessing research gaps and unmet needs in endometriosis. Am. J. Obstet. Gynecol..

[7] Institute for Health Metrics and Evaluation: Endometriosis—Level 4 Cause. http://www.healthdata.org/sites/default/files/disease_and_injury/gbd_2019/topic_pdf/cause/607.pdf.

[8] Ghiasi M., Kulkarni M.T., Missmer S.A. (2020). Is Endometriosis More Common and More Severe Than It Was 30 Years Ago?. J. Minim. Invasive Gynecol..

[9] Saunders P.T.K., Horne A.W. (2021). Endometriosis: Etiology, pathobiology, and therapeutic prospects. Cell.

[10] DiVasta A.D., Vitonis A.F., Laufer M.R., Missmer S.A. (2018). Spectrum of symptoms in women diagnosed with endometriosis during adolescence vs adulthood. Am. J. Obstet. Gynecol..

[11] McPeak A.E., Allaire C., Williams C., Albert A., Lisonkova S., Yong P.J. (2018). Pain Catastrophizing and Pain Health-Related Quality-of-Life in Endometriosis. Clin. J. Pain.

[12] Chapron C., Marcellin L., Borghese B., Santulli P. (2019). Rethinking mechanisms, diagnosis and management of endometriosis. Nat. Rev. Endocrinol..

[13] Shigesi N., Kvaskoff M., Kirtley S., Feng Q., Fang H., Knight J.C., Missmer S.A., Rahmioglu N., Zondervan K.T., Becker C.M. (2019). The association between endometriosis and autoimmune diseases: A systematic review and meta-analysis. Hum. Reprod. Update.

[14] Miller J.A., Missmer S.A., Vitonis A.F., Sarda V., Laufer M.R., DiVasta A.D. (2018). Prevalence of migraines in adolescents with endometriosis. Fertil. Steril..

[15] Montanari G., Di Donato N., Benfenati A., Giovanardi G., Zannoni L., Vicenzi C., Solfrini S., Mignemi G., Villa G., Mabrouk M. (2013). Women with deep infiltrating endometriosis: Sexual satisfaction, desire, orgasm, and pelvic problem interference with sex. J. Sex. Med..

[16] Prescott J., Farland L.V., Tobias D.K., Gaskins A.J., Spiegelman D., Chavarro J.E., Rich-Edwards J.W., Barbieri R.L., Missmer S.A. (2016). A prospective cohort study of endometriosis and subsequent risk of infertility. Hum. Reprod..

[17] Senapati S., Sammel M.D., Morse C., Barnhart K.T. (2016). Impact of endometriosis on in vitro fertilization outcomes: An evaluation of the Society for Assisted Reproductive Technologies Database. Fertil. Steril..

[18] Mu F., Rich-Edwards J., Rimm E.B., Spiegelman D., Forman J.P., Missmer S.A. (2017). Association between Endometriosis and Hypercholesterolemia or Hypertension. Hypertension.

[19] Mu F., Rich-Edwards J., Rimm E.B., Spiegelman D., Missmer S.A. (2016). Endometriosis and risk of coronary heart disease. Circ. Cardiovasc. Qual. Outcomes.

[20] Shafrir A.L., Farland L.V., Shah D.K., Harris H.R., Kvaskoff M., Zondervan K., Missmer S.A. (2018). Risk for and consequences of endometriosis: A critical epidemiologic review. Best Pract. Res. Clin. Obstet. Gynaecol..

[21] Kvaskoff M., Mu F., Terry K.L., Harris H.R., Poole E.M., Farland L., Missmer S.A. (2014). Endometriosis: A high-risk population for major chronic diseases?. Hum. Reprod. Update.

[22] Koninckx P.R., Ussia A., Keckstein J., Wattiez A., Adamyan L. (2016). Epidemiology of subtle, typical, cystic, and deep endometriosis: A systematic review. Gynecol. Surg..

[23] Canis M., Donnez J.G., Guzick D.S., Halme J.K., Rock J.A., Schenken R.S., Vernon M.W. (1997). Revised American Society for Reproductive Medicine classification of endometriosis: 1996. Fertil. Steril..

[24] Lee S.-Y., Koo Y.-J., Lee D.-H. (2021). Classification of endometriosis. Yeungnam Univ. J. Med..

[25] Riazi H., Tehranian N., Ziaei S., Mohammadi E., Hajizadeh E., Montazeri A. (2015). Clinical diagnosis of pelvic endometriosis: A scoping review. BMC Womens. Health.

[26] Parasar P., Ozcan P., Terry K.L. (2017). Endometriosis: Epidemiology, Diagnosis and Clinical Management. Curr. Obstet. Gynecol. Rep..

[27] Viganò P., Somigliana E., Panina P., Rabellotti E., Vercellini P., Candiani M. (2012). Principles of phenomics in endometriosis. Hum. Reprod. Update.

[28] Vercellini P., Eskenazi B., Consonni D., Somigliana E., Parazzini F., Abbiati A., Fedele L. (2011). Oral contraceptives and risk of endometriosis: A systematic review and meta-analysis. Hum. Reprod. Update.

[29] Vercellini P., De Matteis S., Somigliana E., Buggio L., Frattaruolo M.P., Fedele L. (2013). Long-term adjuvant therapy for the prevention of postoperative endometrioma recurrence: A systematic review and meta-analysis. Acta Obstet. Gynecol. Scand..

[30] Farland L.V., Horne A.W. (2019). Disparity in endometriosis diagnoses between racial/ethnic groups. BJOG An Int. J. Obstet. Gynaecol..

[31] Bougie O., Healey J., Singh S.S. (2019). Behind the times: Revisiting endometriosis and race. Am. J. Obstet. Gynecol..

[32] Bougie O., Yap M.I., Sikora L., Flaxman T., Singh S. (2019). Influence of race/ethnicity on prevalence and presentation of endometriosis: A systematic review and meta-analysis. BJOG An Int. J. Obstet. Gynaecol..

[33] Shafrir A.L., Missmer S.A. (2020). Towards subtypes—Deep endometriosis oestrogen receptor-α expression. Nat. Rev. Endocrinol..

[34] Culley L., Law C., Hudson N., Denny E., Mitchell H., Baumgarten M., Raine-Fenning N. (2013). The social and psychological impact of endometriosis on women’s lives: A critical narrative review. Hum. Reprod. Update.

[35] De Graaff A.A., D’hooghe T.M., Dunselman G.A.J., Dirksen C.D., Hummelshoj L., Simoens S., Bokor A., Brandes I., Brodszky V., Canis M. (2013). The significant effect of endometriosis on physical, mental and social wellbeing: Results from an international cross-sectional survey. Hum. Reprod..

[36] Soliman A.M., Coyne K.S., Gries K.S., Castelli-Haley J., Snabes M.C., Surrey E.S. (2017). The effect of endometriosis symptoms on absenteeism and presenteeism in the workplace and at home. J. Manag. Care Spec. Pharm..

[37] Fourquet J., Gao X., Zavala D., Orengo J.C., Abac S., Ruiz A., Laboy J., Flores I. (2010). Patients’ report on how endometriosis affects health, work, and daily life. Fertil. Steril..

[38] Nnoaham K.E., Hummelshoj L., Webster P., D’Hooghe T., De Cicco Nardone F., De Cicco Nardone C., Jenkinson C., Kennedy S.H., Zondervan K.T. (2011). Impact of endometriosis on quality of life and work productivity: A multicenter study across ten countries. Fertil. Steril..

[39] Simoens S., Dunselman G., Dirksen C., Hummelshoj L., Bokor A., Brandes I., Brodszky V., Canis M., Colombo G.L., Deleire T. (2012). The burden of endometriosis: Costs and quality of life of women with endometriosis and treated in referral centres. Hum. Reprod..

[40] Gallagher J.S., DiVasta A.D., Vitonis A.F., Sarda V., Laufer M.R., Missmer S.A. (2018). The Impact of Endometriosis on Quality of Life in Adolescents. J. Adolesc. Health.

[41] Gupta J., Cardoso L.F., Harris C.S., Dance A.D., Seckin T., Baker N., Ferguson Y.O. (2018). How do adolescent girls and boys perceive symptoms suggestive of endometriosis among their peers? Findings from focus group discussions in New York City. BMJ Open.

[42] Fourquet J., Báez L., Figueroa M., Iriarte R.I., Flores I. (2011). Quantification of the impact of endometriosis symptoms on health-related quality of life and work productivity. Fertil. Steril..

[43] Brasil D.L., Montagna E., Trevisan C.M., la Rosa V.L., Laganà A.S., Barbosa C.P., Bianco B., Zaia V. (2020). Psychological stress levels in women with endometriosis: Systematic review and meta-analysis of observational studies. Minerva Med..

[44] Gambadauro P., Carli V., Hadlaczky G. (2019). Depressive symptoms among women with endometriosis: A systematic review and meta-analysis. Am. J. Obstet. Gynecol..

[45] Facchin F., Saita E., Barbara G., Dridi D., Vercellini P. (2018). “Free butterflies will come out of these deep wounds”: A grounded theory of how endometriosis affects women’s psychological health. J. Health Psychol..

[46] Rees M., Kiemle G., Slade P. (2020). Psychological variables and quality of life in women with endometriosis. J. Psychosom. Obstet. Gynecol..

[47] Quinlivan J., Lambregtse-van den Berg M. (2021). Managing the stigma and women’s physical and emotional cost of endometriosis. J. Psychosom. Obstet. Gynecol..

[48] Estes S.J., Huisingh C.E., Chiuve S.E., Petruski-Ivleva N., Missmer S.A. (2021). Depression, anxiety, and self-directed violence in women with endometriosis: A retrospective matched-cohort study. Am. J. Epidemiol..

[49] Barbara G., Facchin F., Meschia M., Berlanda N., Frattaruolo M.P., Vercellini P. (2017). When love hurts. A systematic review on the effects of surgical and pharmacological treatments for endometriosis on female sexual functioning. Acta Obstet. Gynecol. Scand..

[50] Barbara G., Facchin F., Buggio L., Somigliana E., Berlanda N., Kustermann A., Vercellini P. (2017). What Is Known and Unknown About the Association Between Endometriosis and Sexual Functioning: A Systematic Review of the Literature. Reprod. Sci..

[51] Van Niekerk L.M., Schubert E., Matthewson M. (2021). Emotional intimacy, empathic concern, and relationship satisfaction in women with endometriosis and their partners. J. Psychosom. Obstet. Gynecol..

[52] Corte L.D., Di Filippo C., Gabrielli O., Reppuccia S., La Rosa V.L., Ragusa R., Fichera M., Commodari E., Bifulco G., Giampaolino P. (2020). The burden of endometriosis on women’s lifespan: A narrative overview on quality of life and psychosocial wellbeing. Int. J. Environ. Res. Public Health.

[53] Bernuit D., Ebert A.D., Halis G., Strothmann A., Gerlinger C., Geppert K., Faustmann T. (2011). Female perspectives on endometriosis: Findings from the uterine bleeding and pain women’s research study. J. Endometr..

[54] Hudelist G., Fritzer N., Thomas A., Niehues C., Oppelt P., Haas D., Tammaa A., Salzer H. (2012). Diagnostic delay for endometriosis in Austria and Germany: Causes and possible consequences. Hum. Reprod..

[55] Goffman E. (1963). Notes on the Management of Spoiled Identity.

[56] Link B.G., Phelan J.C. (2001). Conceptualizing Stigma. Annu. Rev. Sociol..

[57] Elliott G.C., Ziegler H.L., Altman B.M., Scott D.R. (1982). Understanding stigma: Dimensions of deviance and coping. Deviant Behav..

[58] Crocker J., Major B., Steele C., Gilbert D.T., Fiske S.T., Lindzey G. (1988). Social Stigma. The Handbook of Social Psychology.

[59] Scambler G. (1998). Stigma and disease: Changing paradigms. Lancet.

[60] Lucksted A., Drapalski A.L. (2015). Self-Stigma Regarding Mental Illness: Definition, Impact, and Relationship to Societal Stigma. Psychiatr. Rehabil. J..

[61] Phelan J.C., Link B.G., Dovidio J.F. (2008). Stigma and prejudice: One animal or two?. Soc. Sci. Med..

[62] Stuber J., Meyer I., Link B. (2008). Stigma, prejudice, discrimination and health. Soc. Sci. Med..

[63] Hatzenbuehler M.L., Phelan J.C., Link B.G. (2013). Stigma as a fundamental cause of population health inequalities. Am. J. Public Health.

[64] Ahern J., Stuber J., Galea S. (2007). Stigma, discrimination and the health of illicit drug users. Drug Alcohol Depend..

[65] Reif S., Wilson E., McAllaster C., Pence B. (2019). The Relationship of HIV-related Stigma and Health Care Outcomes in the US Deep South. AIDS Behav..

[66] Arnold E.A., Rebchook G.M., Kegeles S.M. (2014). “Triply cursed”: Racism, homophobia and HIV-related stigma are barriers to regular HIV testing, treatment adherence and disclosure among young Black gay men. Cult. Health Sex..

[67] McCoy K., Lipira L., Kemp C.G., Nevin P.E., Huh D., Turan J.M., Mugavero M.J., Cohn S.E., Bahk M., Simoni J.M. (2020). Exploring HIV-Related Stigma as a Determinant of Engagement in HIV Care by African American Women. J. Assoc. Nurses AIDS Care.

[68] Marinho R.T., Barreira D.P. (2013). Hepatitis C, stigma and cure. World J. Gastroenterol..

[69] Wu Y.K., Berry D.C. (2018). Impact of weight stigma on physiological and psychological health outcomes for overweight and obese adults: A systematic review. J. Adv. Nurs..

[70] Olesen K., Cleal B., Willaing I. (2020). Discrimination and stigma among people with type 2 diabetes in the workplace: Prejudice against illness or obesity?. Public Health.

[71] Glynn T.R., Llabre M.M., Lee J.S., Bedoya C.A., Pinkston M.M., O’Cleirigh C., Safren S.A. (2019). Pathways to Health: An Examination of HIV-Related Stigma, Life Stressors, Depression, and Substance Use. Int. J. Behav. Med..

[72] Hawke L.D., Parikh S.V., Michalak E.E. (2013). Stigma and bipolar disorder: A review of the literature. J. Affect. Disord..

[73] Chesney M.A., Ickovics J.R., Chambers D.B., Gifford A.L., Neidig J., Zwickl B., Wu A.W. (2000). Self-reported adherence to antiretroviral medications among participants in HIV clinical trials: The AACTG adherence instruments. Patient Care Committee & Adherence Working Group of the Outcomes Committee of the Adult AIDS Clinical Trials Group (AACTG). AIDS Care.

[74] Matías-González Y., Sánchez-Galarza A.N., Flores-Caldera I., Rivera-Segarra E. (2021). “Es que tú eres una changa”: Stigma experiences among Latina women living with endometriosis. J. Psychosom. Obstet. Gynecol..

[75] Seear K. (2009). The etiquette of endometriosis: Stigmatisation, menstrual concealment and the diagnostic delay. Soc. Sci. Med..

[76] Elle Saine M., Moore T.M., Szymczak J.E., Bamford L.P., Barg F.K., Mitra N., Schnittker J., Holmes J.H., Lo Re V. (2020). Validation of a modified Berger HIV stigma scale for use among patients with hepatitis C virus (HCV) infection. PLoS ONE.

[77] Van Rie A., Sengupta S., Pungrassami P., Balthip Q., Choonuan S., Kasetjaroen Y., Strauss R.P., Chongsuvivatwong V. (2008). Measuring stigma associated with tuberculosis and HIV/AIDS in southern Thailand: Exploratory and confirmatory factor analyses of two new scales. Trop. Med. Int. Health.

[78] Browne J.L., Ventura A.D., Mosely K., Speight J. (2017). Measuring Type 1 diabetes stigma: Development and validation of the Type 1 Diabetes Stigma Assessment Scale (DSAS-1). Diabet. Med..

[79] Yang L.H., Grivel M.M., Anderson B., Bailey G.L., Opler M., Wong L.Y., Stein M.D. (2019). A new brief opioid stigma scale to assess perceived public attitudes and internalized stigma: Evidence for construct validity. J. Subst. Abuse Treat..

[80] Schofield C.A., Ponzini G.T. (2020). The Skidmore Anxiety Stigma Scale (SASS): A covert and brief self-report measure. J. Anxiety Disord..

[81] Coughlin S.S. (2015). “Test, Listen, Cure” (TLC) Hepatitis C Community Awareness Campaign. JMIR Res. Protoc..

[82] Hagan L.M., Schinazi R.F. (2013). Best strategies for global HCV eradication. Liver Int..

[83] Maticic M., Pirnat Z., Leicht A., Zimmermann R., Windelinck T., Jauffret-Roustide M., Duffell E., Tammi T., Schatz E. (2020). The civil society monitoring of hepatitis C response related to the WHO 2030 elimination goals in 35 European countries. Harm Reduct. J..

[84] Makita M., Mas-Bleda A., Morris S., Thelwall M. (2021). Mental Health Discourses on Twitter during Mental Health Awareness Week. Issues Ment. Health Nurs..

[85] Livingston J.D., Tugwell A., Korf-Uzan K., Cianfrone M., Coniglio C. (2013). Evaluation of a campaign to improve awareness and attitudes of young people towards mental health issues. Soc. Psychiatry Psychiatr. Epidemiol..

[86] Cocksedge K.A., Guliani J., Henley W., Anderson T., Roberts S., Reed L., Skinnard D., Fisher S., Chapman B., Willcox J. (2019). Local radio to promote mental health awareness: A public health initiative. BJPsych Open.

[87] Kerr J., Ayangeakaa S., Combs R., Harris L., Sears J., Northington T., Burton K., Sterrett-Hong E., Parker K., Krigger K. (2020). Community-Informed Development of a Campaign to Increase HIV Pre-exposure Prophylaxis (PrEP) Awareness Among African-American Young Adults. J. Racial Ethn. Health Disparities.

[88] Centers for Disease Control and Prevention World AIDS Day—December 1. https://www.cdc.gov/hiv/library/awareness/wad.html.

[89] Cook J.E., Purdie-Vaughns V., Meyer I.H., Busch J.T.A. (2014). Intervening within and across levels: A multilevel approach to stigma and public health. Soc. Sci. Med..

